# Exposure to Lymphocytic Choriomeningitis Virus, New York, USA

**DOI:** 10.3201/eid1707.101349

**Published:** 2011-07

**Authors:** Barbara Knust, Adam MacNeil, Susan J. Wong, P. Bryon Backenson, Aridth Gibbons, Pierre E. Rollin, Stuart T. Nichol

**Affiliations:** Author affiliations: Centers for Disease Control and Prevention, Atlanta, Georgia, USA (B. Knust, A. MacNeil, A. Gibbons, P.E. Rollin, S.T. Nichol);; New York State Department of Health, Albany, New York, USA (S.J. Wong, P.B. Backenson)

**Keywords:** lymphocytic choriomeningitis virus, seroepidemiologic studies, enzyme-linked immunosorbent assay, New York, viruses, letter

**To the Editor:** Lymphocytic choriomeningitis virus (LCMV) is an arenavirus carried by the house mouse, *Mus musculus*. Human infections can range from mild febrile illness to severe encephalitis and disseminated disease (*1*). Infection during pregnancy is associated with teratogenic effects, including congenital hydrocephalus and chorioretinitis (*2*).

The overall occurrence of human exposure to LCMV is not known. Two large US serosurveys suggest that 3%–5% of persons tested had previous LCMV exposure as measured by immunoglobulin (Ig) G (*3**,**4*). In 2002, LCMV-associated congenital subependymal calcifications, hydrocephalus, and chorioretinitis were confirmed for 2 children in central Syracuse, Onondaga County, New York, USA. In 2009, the Centers for Disease Control and Prevention confirmed another case of LCMV-associated congenital hydrocephalus and chorioretinitis in a child from the same neighborhood. For each of the 3 cases, the mother’s history included exposure to mice during pregnancy. One mother also had a pet guinea pig, which had negative results for LCMV by serologic testing and reverse transcription PCR of kidney tissue (*5*).

Congenital LCMV is rarely reported to public health departments or in the literature. Therefore, to better understand the magnitude of LCMV exposure in the general population of Onondaga County, we conducted a serosurvey. The American Red Cross provided the Wadsworth Center of the New York State Department of Health with blood or serum samples collected from persons >16 years of age at blood drives during August 2009. Information about date of birth, sex, and county and ZIP code of residence was provided. A subset of samples from blood donors residing in Onondaga County were tested at the Centers for Disease and Prevention by ELISA for LCMV IgM and IgG as described (*4*). State and federal institutional review board approval was obtained for this study.

Samples from 562 blood donors were tested. Mean age of donors was 48 years (median 50 ± 15 SD, range 17–79 years). LCMV IgG was detected in 2 (0.4%) samples (titer >400) and was undetectable in all other samples. LCMV IgM was not detected in any samples. Of the 25 donors who reported residing in 1 of the 2 ZIP codes as the case-patients with congenital LCMV, none had positive test results.

Given our findings, little evidence supports a high level of human exposure to LCMV in Onondaga County. Compared with previously reported seroprevalences of 3%–5%, the proportion of persons exposed to LCMV was lower than expected (*3**,**4*). The same serologic assay was used in this study and the 2 previous US serosurveys, suggesting that the different results are not an artifact of different assays. Additionally, persons tested in the current survey were older than those tested in previous serosurveys (median 50 vs. 23 [*3*] and 40 years [*4*], respectively). Because IgG against LCMV can persist for years, seroprevalence would be expected to be higher for an older population as a result of more chances for exposure. Also, a serosurvey of >1,000 hospitalized persons from upstate New York in the 1970s detected no positive antibody titers (*6*), consistent with our findings.

Our serosurvey had a few limitations. Blood samples from an entire county cannot detect potential household- or neighborhood-scale areas of increased risk for LCMV exposure, which may be related to focal distribution of populations of LCMV-infected house mice. Serosurveys of house mice have previously shown evidence for clustering of LCMV-infected individuals (*7*); however, the prevalence of LCMV in house mice in Onondaga County is unknown. Additionally, because blood donors were volunteers, the population sampled did not necessarily reflect the population at risk for LCMV exposure. Despite these considerations, the low prevalence of LCMV antibodies suggests low occurrence of LCMV exposure in this population.

Although little is known about frequency of human exposure and infection, LCMV seems to be rare with a propensity for inducing severe disease. LCMV infection has been associated with high incidence of clinical disease, including a pet hamster–associated outbreak in 1973–1974 that resulted in at least 181 cases and 46 hospitalizations in 12 states (*8*).

LCMV-related disease is reportable in only 3 states (Wisconsin, Massachusetts, Arizona) and 1 city (New York, New York) and is considered to be widely undertested and underdiagnosed. A recent survey of health care providers in Connecticut found that LCMV diagnostic tests were not requested for all patients suspected to have LCMV infection (*9*); thus, missed diagnoses are possible. Additional studies are needed to understand the incidence of LCMV-related disease and LCMV seroprevalence in the general population (*10*).
